# A machine learning language model approach to evaluating mental health awareness content across Spanish- and English-language social media posts on Twitter

**DOI:** 10.1007/s00127-025-02870-y

**Published:** 2025-03-06

**Authors:** Melissa J. DuPont-Reyes, Wenxue Zou, Jinxu Li, Alice P. Villatoro, Lu Tang

**Affiliations:** 1 Department of Sociomedical Sciences, Columbia University Mailman School of Public Health, New York, NY USA; 2 Department of Epidemiology, Columbia University Mailman School of Public Health, 722 West 168th Street, New York, NY 10032 USA; 3 Department of Communication, Coastal Carolina University, Conway, SC USA; 4 Department of Communication and Journalism, Texas A&M University, College Station, TX USA; 5 Department of Public Health, Santa Clara University, Santa Clara, CA USA

**Keywords:** Mental health awareness, Social media, Spanish, Stigma, Twitter, X

## Abstract

**Purpose:**

Mental health information appears on social media in varying levels of quality and may or may not be productive information to users, particularly in relation to healthcare decision-making and community living among diverse populations coping with mental health problems. To better understand the mental health landscape on social media, this study validated a language model approach to evaluating the availability and sentiment of mental health awareness content across Spanish- and English-language social media posts on Twitter (currently X) to inform future mental health communication guidelines.

**Methods:**

A comprehensive list of mental health awareness hashtags in Spanish and English was developed by bilingual investigators to download tweets containing these hashtags in both languages from the Twitter Academic API from 09/19/22 − 10/10/22. Data extraction and cleaning of duplicate tweets resulted in a final sample of 28,268 Spanish and 205,774 English tweets for sentiment and structural topic analysis across the two languages.

**Results:**

Fifteen unique topics emerged for both Spanish and English tweets including overlapping themes of awareness, self-care, lived experience, and service providers. Topics in Spanish tweets were more often significantly associated with negative emotions compared to English tweets. Yet English tweets also included misappropriation of mental health labels to make political statements and market products.

**Conclusions:**

Mental health awareness content on Twitter appears not to be consistently available or aligned with clinical values, disadvantaging Spanish-language social media users, possibly leading to divergent priorities concerning population mental health. Nevertheless, natural language processing techniques offers a viable method to further understand unequal mental health awareness content across various language and cultural social media.

## Introduction

A growing body of research suggests that the United States is facing a population mental health crisis, influenced by unfavorable social circumstances among persons with intersecting social risk factors (e.g., poverty and race/ethnic minoritized groups) and later exacerbated during the COVID-19 pandemic [1–3]. Concurrently, social media has emerged as an accessible source of health information with swift and broad reach, including mental health information. The use of social media has become almost ubiquitous, increasing users’ exposure to mental health awareness content such as information about mental health symptom management and peer support [4]. However, there may also be mental health risks associated with exposure to mental health content on social media such as encountering pejorative, stigmatizing mental health messaging, cyberbullying, overconcern about body image, excessive peer comparison, and hate speech [5–9]. Nevertheless, mental health awareness content in social media may positively shape mental health by delivering anti-stigma content, enhancing mental health self-perceptions including the identification of a potential mental health problem, and promoting help-seeking beliefs and behaviors including knowledge of accessible resources to receive support. Indeed long-standing theoretical traditions regarding mental health stigma emphasize the influence of social networks on mental health outcomes and the importance of anti-stigma messaging on healthy behavioral outcomes for those coping with mental illness [10, 11]. These processes may also be occurring in the social media landscape.

As a valuable source of feedback for researchers aiming to understand mental health awareness content on social media, Twitter (now currently X) can be used to investigate real-time diffusion of mental health awareness discussions on social media to understand the collective thoughts and sentiments of a diverse user base concerning mental health [4, 12]. Identifying trends and patterns in mental health awareness content on Twitter can help our understanding of the range and quality of mental health awareness content that the public may be exposed to, which can influence their health behavior and health care decision-making. However, comprehensive insights into mental health awareness content in social media, especially in various language and cultural contexts such as Spanish-language social media, have often been overlooked in research [13, 14]. While researchers have focused on various health topics on Spanish-language Twitter, including vaccine uptake/hesitancy related to COVID-19, HIV, Mpox, and HPV, mental health has received comparatively less attention [15–19]. Still some studies of Spanish-language Twitter that have been conducted outside of the United States have focused on depression and anorexia, though these studies did not have a comparator group or dataset of English-language Twitter [20–23].

Nevertheless, prior survey-based studies among Latino residents in the United States has identified higher exposure of mental health promotion content in English-language media compared to Spanish/Latino media i.e., media in Spanish-language and/or tailored to Latino audiences [24]. These disparities are posited to be predominantly due to health inequity-producing processes whereby the diffusion of mental health communication campaigns reach marginalized populations less often and slower, if at all [13, 25]. Consequently, Spanish/Latino media may contain elevated levels of mental health pejorative content [13, 24, 25]. Moreover, Latino populations in the United States exhibit higher social media use than other race/ethnic groups and are impacted by distinct mental health messaging within their various language and cultural social media landscape [13, 25–28]. Spanish is also the most common non-English language spoken in the United States, with the country boasting the title of having the second-largest population of Spanish speakers worldwide following Mexico. Therefore, it is imperative to examine how mental health awareness content may vary across different languages on Twitter. This exploration can inform future guidelines concerning mass communication about mental health towards holding a more global perspective concerning various language and cultural social media in emerging technologies.

The current study extends prior studies in the field to assess mental health awareness content *comparatively* across Spanish- and English-language Twitter during the same data collection period. We anticipate discovering a greater availability of mental health awareness content in English-language Twitter compared to its Spanish-language counterpart. Furthermore, we expect the English-language content to exhibit a generally higher quality and positive reception than Spanish-language Twitter. In this study, we employ machine learning techniques, specifically natural language process programs, to analyze various language and cultural Twitter data, thereby also validating this tool for examining effectiveness of mental health campaigns in the future. This analysis aims to increase our global understanding of the social media landscape with respect to mental health awareness content, and further fairness and accountability in the use of technology for population mental health.

## Methods

### Data collection and sampling

Twitter provided a rich source of data on user experiences and opinions, and was readily available for researchers to examine, making it a valuable platform for the study of mental health awareness content in social media. First, we compiled a comprehensive list of hashtags related to mental health awareness in both Spanish-language and English-language, resulting in 16 hashtags in each language (Table 1). Next, Twitter Academic API was used to download all relevant Twitter data containing the 16 Spanish-language and English-language hashtags over a 22-day period (09/19/22 − 10/10/22) using a Python script; all data was collected in the period prior to the acquisition of Twitter by Elon Musk, who then rebranded the social media company as X. A total of 123,858 original tweets using the mental health Spanish-language hashtags and 815,401 original tweets using the English-language mental health hashtags were gathered. Duplicate tweets were removed, resulting in a final analytical dataset of 28,268 Spanish-language tweets and 205,774 English-language tweets.

**Table 1:** List of English- and Spanish-Language Twitter hashtags for Twitter academic API search from 09/19/22 − 10/10/22

Twitter English #	Twitter Spanish #
#mentalhealth	#saludmental
#anxiety	#ansiedad
#depression	#depresión
#wellbeing	#bienestar
#mentalillness	#enfermedadmental
#stress	#estrés
#psychologist	#psicólogo
#selfesteem	#autoestima
#mentalhealthmatters	#lasaludmentalimporta
#MentalHealthMonth	#mesdesaludmental
#schizophrenia	#esquizofrenia
#bipolar	#Bipolar
#psychosis	#psicosis
#mentalhealthstigma	#estigmadesaludmental
#breakthestigma	#rompeelestigma
#postpartumdepression	#DepresionPostparto

### Sentiment analysis

Sentiment analysis (SA) or opinion mining is a subfield within natural language processing (NLP) that involves the computational processing of opinions, feelings, and subjectivity in text data [29]. To analyze sentiment in our Twitter sample, we utilized the English and Spanish dictionaries provided by the Linguistic Inquiry and Word Count software (LIWC2007). LIWC2007 employs an internal default dictionary to determine which words should be considered in the target text file [30]. The dictionary categorizes phrases for specific domains, including negative sentiment words, referred to as sub-dictionaries or word classes [30]. Although LIWC2007 is a relatively older version, it is the latest version of a dictionary available in both English and Spanish. LIWC2007 was previously utilized for sentiment analysis on public discussions regarding health issues on social media, both in English and Spanish, covering various topics such as infectious diseases, vaccine discussions, and depression assessment [19]. Within LIWC2007, positive and negative emotions measure emotion and comprise 406 and 499 words, respectively [30]. Sentiment scores were calculated by subtracting the negative tone from the positive tone to determine the overall emotional value of each tweet. Scores greater than 0 indicated a positive sentiment and scores less than 0 indicated a negative sentiment, while scores equal to 0 categorized the sentiment as neutral.

### Structural topic modeling analysis

Structural topic modeling (STM) which is an unsupervised learning technique was also applied to text data for analysis. Similar to other topic models like Latent Dirichlet Allocation (LDA) and Correlated Topic Model (CTM), STM is a generative model used to analyze word counts in text data [31]. This approach involves defining a data-generating process for each tweet and utilizing available data to determine the most probable parameter values within the model [31]. STM utilizes a statistical model to generate a tweet-topic probability matrix, which estimates the probability of each tweet belonging to different topics. Building upon the foundation of probabilistic topic models, STM extends these models to incorporate covariates of interest in the prior distributions of document-topic proportions and topic-word distributions [32]. STM has found wide application in the analysis of social media content including public health topics.

To examine the prevalent topics related to mental health awareness and their corresponding sentiments, we utilized sentiment metadata (negative/positive) in the STM to estimate topic proportions within the Spanish and English Twitter datasets separately. Due to the binary nature of the covariates allowed by the STM (negative or positive), neutral sentiment tweets in the sample were not analytically tested by the STM. Multiple candidate models (i.e., 5 to 30 topic clusters) were tested to identify the most suitable number of clusters. After considering various quantitative metrics such as held-out likelihood, residual, and semantic coherence, as well as conducting manual reviews by the authors to assess the degree of thematic overlaps among topics, we selected the 15-topic model for further analysis. To analyze the thematic characteristics of each topic, we identified four indicators of top words, including high probability, FREX, lift, and score^1^. However, for the sake of brevity, we only included the high-probability words in Tables 2 and 3. Subsequently, we grouped similar topics together under a common overarching theme by considering their semantic similarities.

**Table 2:** Topics discussed in Spanish-Language Twitter sample, Twitter academic API search from 09/19/22 − 10/10/22

Theme	Topic Number	High-Probability Words	Sample Tweet
Awareness	2. Intimate partnerships and mental health	ansiedad, estrés, depresión, terapia, psicología, miedo, depresión	3 razones comunes por las que las parejas se desenamoran #pareja #desamor #relacion #psicologia #PSICAUDIO #podcast #mente #conducta #comportamiento #audio #emocion #sujeto #estudio #ansiedad #educacion #estres #trastorno #personalidad
	4. Youth mental health	saludmental, persona, depresión, problema, trastorno, enfermedad, suicidio	RT @Agifes La #saludmental de los adolescentes está en crisis. El suicidio es la cuarta causa de muerte entre los jóvenes de 15 a 19 años, y que 1 de cada 7 jóvenes de entre 10 y 19 años padece algún tipo de trastorno mental @mntemaravillosa
	5. Mental health advocacy	atención, diamundialdelasaludmental, prevención, derecho, día mundial de la saludmental, social, persona	RT @Defensoria_Peru El Estado debe garantizar #DerechoALaSaludMental con una atención oportuna, de calidad y accesible. Desde la sociedad civil debemos contribuir a eliminar estigmas que reprimen a personas de acudir a servicios de #SaludMental. Nos sumamos a la campaña de @opsoms #HazTuParte.
	6. World mental health day	salud, mental, saludment, mundial, hoy, importancia, octubr	@trabemsoftware Hoy 10 de octubre se conmemora el Día Mundial de la Salud Mental, una efeméride impulsada por la Federación Mundial para la Salud Mental (WFMH) con el apoyo de la Organización Mundial de la Salud (OMS). #saludmental #diamundial #softwareclinica #salud #ehealth
	9. Welfare advocacy for mental illness	gent, ser, esquizofrenia, colombia, mucha, hoy, permanent	RT @con_menudo @sanidadgob @educaciongob @UniversidadGob HAZ MAGIA TWITTER @sanidadgob @inclusiongob @CarolinaDarias @joseluisescriva Para cuando una investigación en Sevilla, C/Sánchez Perrier N2 sede del INSS. No se concede ni una sola incapacidad permanente absoluta por #SaludMental, luego derivan en #suicidio.
	10. Mental health services advocacy	bien, pedir, ayuda, necesitan, profesion, buscar, sevilla	RT @con_menudo @incluinfo 20* día de espera, el INSS de Sevilla siguen estudiando la reclamación previa, nosotros velando por qué mí hermano este bien las 24 h. #SaludMental #suicidio Señor Fernando Camilo, está usted muy ocupado? O ya ha salido del trabajo?@joseluisescriva
Self-care	1. Self-care and well-being	bienestar, salud, desarrollo, saludemocion, felicidad, empresa, cuida	RT @TerapiasKi… y observa la magia. ¡Sigamos con fe la semana amado universo! #Espiritualidad #SaludEmocional #PazInterior #Gratitud #Chakras #AmorPropio #Bienestar #MantrasdeLuz #Magia #UnEsfuerzoMás
	3. Astrology and well-being	autoestima, mujer, bienestar, puede, signosdelzodíaco, felizlun, astrología	RT @elhoroscopodel1 #Libra en ocasiones, es necesario que recuerdes que no se vive eternamente. Así que ve a por tus sueños. #Horóscopo #SignosdelZodíaco #Autoestima #FelizMiércoles. Si te gustó dale al Síguenos para más
	13. Mindfullness self-care	vida, bienestar, emoción, amor, mejor, ser, tener	Tú marcas el ritmo, son tuyos los pasos #BuenosDías #EligeSerFeliz #Reflexiones #Frases #Vida #Actitud #CrecimientoPersonal #Fluir #Responsabilidad #Aceptación #AmorPropio #Consciencia #Madurez #Creencias #Autoconocimiento #Pensamientos #Calma #Bienestar
	15. Fostering resilience	saludment, feliz, bienestar, dentro, energía, felicidad, gran	¡Feliz Jueves Energía Creadora! #elisabetponsa ¿Quieres aprender herramientas para mejorar tu autoestima y felicidad? ¡Levanta el dedo y únete a nuestro taller con @elisabetponsa! #felicidad #autoayuda #saludnatural #nutrigenómica #TeamHope #hopers #construyendofelicidadtodoslosdías #autoayudaemocional
Lived Experience	7. Referrals to blogs/books	consejo, salud, cuerpo, blog, saludment, mejor, sistema	Yelitza Salas: ¿Es tu salud tu mejor activo? …. MI CONSEJO FINAN … #blog #blogger #SaludMental #Salud #descanso #inversion #neuroscience #entrenamiento #Psicologia #Covid_19 #2Nov #finanzaspersonales #COVID19 #FelizViernes #VidaModerna
	11. Promotion of memoirs	saludment, psicología, gracia, mayor, ansiedad, nuevo, español	1. Mi historia. Por Ernesto Cruz T. Nuevo contenido Sólo en I’M HERE, el nuevo blog de … La mayor web en español especializada en Psicología y Ansiedad #Ansiedad #SaludMental
	12. Mental health support group	saludment, semana, octubre, hora, lunes, grupo, experiencia	!!Bonito martes abejitas!!!Flexibilidad! @Crypto_Swarm @SBU_DAO_Espanol #Web3 #NFT #sbudao #SaludMental
Service Providers	8. Mental health clinic	saludment, trabajo, jornada, bienestar, labor, actividad, seguridad	RT @MenniNavarra VIII JORNADA DE SEGURIDAD Y GESTIÓN DEL RIESGO EN #SALUDMENTAL Organizada por @MenniNavarra en colaboración con @JanssenESP y @qna_excelencia. #MenniNavarra #MenniNafarroa Previa #INSCRIPCIÓN en el siguiente enlace.
N/A (Removed)	14. Efforts for animal rights	bienestar, familia, programa, gracia, gobierno, cuenta, apoyo	@MhefisT #tito tiene #Familia, no fue #abandono, sino que huyó con su hermana #tita tras #accidente grave coche, es muy querido y es #miembrodefamilia y se le buscó sin descanso desde el primer momento y con muchos recursos RESPETAR sus #derechosanimales y como #SerSintiente velar #bienestar

**Table 3:** Topics discussed in English-Language Twitter sample, Twitter academic API search from 09/19/22 − 10/10/22

Theme	Topic Number	High-Probability Words	Sample Tweet
Awareness	6. PTSD and suicide awareness	never, someon, anyth, els, lose, alon, hurt	Whether it’s this, #suicide, #depression, #PTSD, the list is never ending… we definitely do not “beat around the bush” and will not use “safe words.” Thank you, and hopefully more people will start talking and doing something about all of these problems in the World.
	7. World mental health day	health, mental, worldmentalhealthday, day, world, today, import	10 October is World Mental Health Day. “The overall objective of World Mental Health Day is to raise awareness of mental health issues around the world and to mobilize efforts in support of mental health” WHO, 2020 #depression #mentalHealthChallenge @G4G_Africa @PaintedBraiNews
	12. Comorbidity (e.g., trauma, pain, substance abuse, cardiovascular disease)	depress, can, suicid, disord, problem, treatment, caus	#PrenatalAlcoholExposure a major cause of #FASD harm to brain development, resulting in vulnerability, risk, development of #BehavioralHealth problems, including #anxiety #depression #suicidality #OpioidUseDisorders #violence #trauma #BrainFunction #CausalityCrisis #SharedFacts
Self-care	3. Faith and wellbeing	thank, great, well, week, join, share, event	HOLY HOLY HOLY #DeliverUsJesus #WithGod #PowerPlatform #Jesus #BlackTwitter #BlackTechTwitter #Holy #Knowledge #WellBeing #Wellness #CyberSecurity #Motivation #Faith #Love #God #Faith #InTheSpirit #Humility #PeacePlease
	4. Mental health self-care	creat, social, learn, wellb, workplac, communiti, educ	Taking care of #mentalhealth is not only vital for overall well-being, but it can impact business as well. Here are three quick tips for managing your mental health and stress in the #workplace: #companyculture
	11. Mindfulness self-care	mind, love, life, happi, selfcar, inspir, heal	RT @KariJoys Loving #Kindness heals the #Heart! #JoyTrain #Joy #Love #Peace #Blessed #IDWP #MentalHealth #Mindfulness #GoldenHearts #IAM #Quote #Blessed #IQRTG #Quotes #SuperSoulSunday #SundayMorning #SundayThoughts #SundayMotivation #ThinkBigSundayWithMarsha
	14. Workplace mental health	work, manag, job, employe, team, peopl, need	When things feel overwhelming, start where you are and figure out the single best next step you can take. Illustration and caption by @fosslien #NextStep #Focus #Anxiety #MentalHealthAwareness #MentalHealthMatters #work #workplace #Motivational #Wellbeing #Health #WorkCulture
	15. Fostering resilience	can, help, best, find, way, start, get	“Out of suffering have emerged the strongest souls; the most massive characters are seared with scars.” -Khalil Gibran #emotionalguidancecoach #injoyinmyself #pattiemartins #thetruetrue #emotionalintelligence #emotionalintelligencecoach #mentalhealth #Counseling #onlinetherapy
Lived Experience	8. Referrals to blogs/books	book, read, free, visit, new, blog, pleas	Visit my #blog for some exciting #articles #Makeup #Beauty, #depression #LifeWithCerebralPalsy
	13. Promotion of memoirs	live, now, podcast, stori, play, episod, listen	@AuthorJRose Thanks Jupiter. My life story #Soundtracktoalife last chapter happening now in real-time.#TrueStory #book #film #ChildSexAbuse #football #prison #mentalhealth #family #seekingjustice #Closure From Left Wing to B Wing
Service Providers	2. Support for mental health service providers	support, servic, care, student, provid, children, need	RT @IOMEthiopia IOM is committed to strengthening the capacity of relevant #MentalHealth & Psychosocial Support (MHPSS) services provided to migrants. In August and September, IOM conducted MHPSS training to 154 service providers assisting vulnerable Ethiopian migrant returnees.
Political Activism	5. Mental illness labels for protest	feel, can, like, emot, peopl, help, parent	#PSYCHOPATHS: NO morals, NO empathy, NO shame + NO consciences! #pathocracy #tyranny #fascism #NarcissisticCollusion #psychopathology #pathology #sociopaths #sadists #CrueltyIsThePoint 7yrs of ACCURATE warnings by 1000s of #MentalHealth Experts on trump, gop, cult 45 etc!
	9. Promotion of white papers	busi, human, video, truth, growth, nft, trend	#Transparentdemocracies #Democracy #Truth #Mainstreammedia #Business #Politics #Democracynow #Mentalhealth #Wellbeing #Foryou #Foryourfamily #Starsflightlimited #Nft #Nftdrop #Transparency #Growth #Humanity
Marketing	1. Mental illness labels for marketing	anxieti, stress, badg, gift, awar, health, hand	Lepidolite, Black Obsidian Bracelet, 8 mm Gemstone Bracelets, Anxiety Calming Stretch Bracelet, Purple Bracelet, Spiritual Crystal Bracelet #black #Bracelets #Obsidian #Stretch #Gemstone #Braceletpurple #Anxiety #Calming #LuckyGem7 #EtsySeller
	10. Complementary and alternative medicine	relax, sleep, natur, medit, well, walk, fit	HempMeds CBD Gummies 1200 mg #CBD #CBDa #Cannabidiol #HempMeds #HempOils #USHemp #HempAuthority #Certified #ShopCBD #Hemp #CBN #MyHealthIsBetter #Pickleball #Anxiety #pain #CBDmedicine

## Results

### Mental health Twitter hashtags: positive, negative, or neutral?

The Spanish language Twitter dataset of 28,268 tweets included 40% (*n* = 11,336) positive, 22% (*n* = 6,064) negative, and 38% (*n* = 10,868) neutral tweets. In contrast, among the 205,774 English tweets analyzed 55% were positive (*n* = 113,173), 18% (*n* = 37,059) were negative, and 27% (*n* = 55,542) were neutral sentiment tweets.

### Themes across Spanish-language mental health Twitter hashtags

Four overarching themes across the 15 topics emerged among the Spanish-language tweets: awareness, self-care, lived experience, and service providers. See Table 2 for high-probability words and example tweets for each of the 15 topics. The awareness theme was the most prominent and encompassed six topics, including intimate partnerships and mental health (Topic 2), youth mental health (Topic 4), mental health advocacy (Topic 5), World Mental Health Day (Topic 6), welfare advocacy for mental illness (Topic 9), and mental health services advocacy (Topic 10). Within the self-care theme, we observed topics such as self-care and well-being (Topic 1), astrology and well-being (Topic 3), mindfulness self-care (Topic 13), and fostering resilience (Topic 15). The lived experience theme included topics related to referrals to blogs/books (Topic 7), promotion of mental health memoirs (Topic 11), and mental health support groups (Topic 12). Lastly, the service providers theme primarily included information about mental health clinics (Topic 8). Notably, Topic 14 was about animal rights and was deemed irrelevant to our study and therefore excluded from further interpretations.

**Fig. 1:**
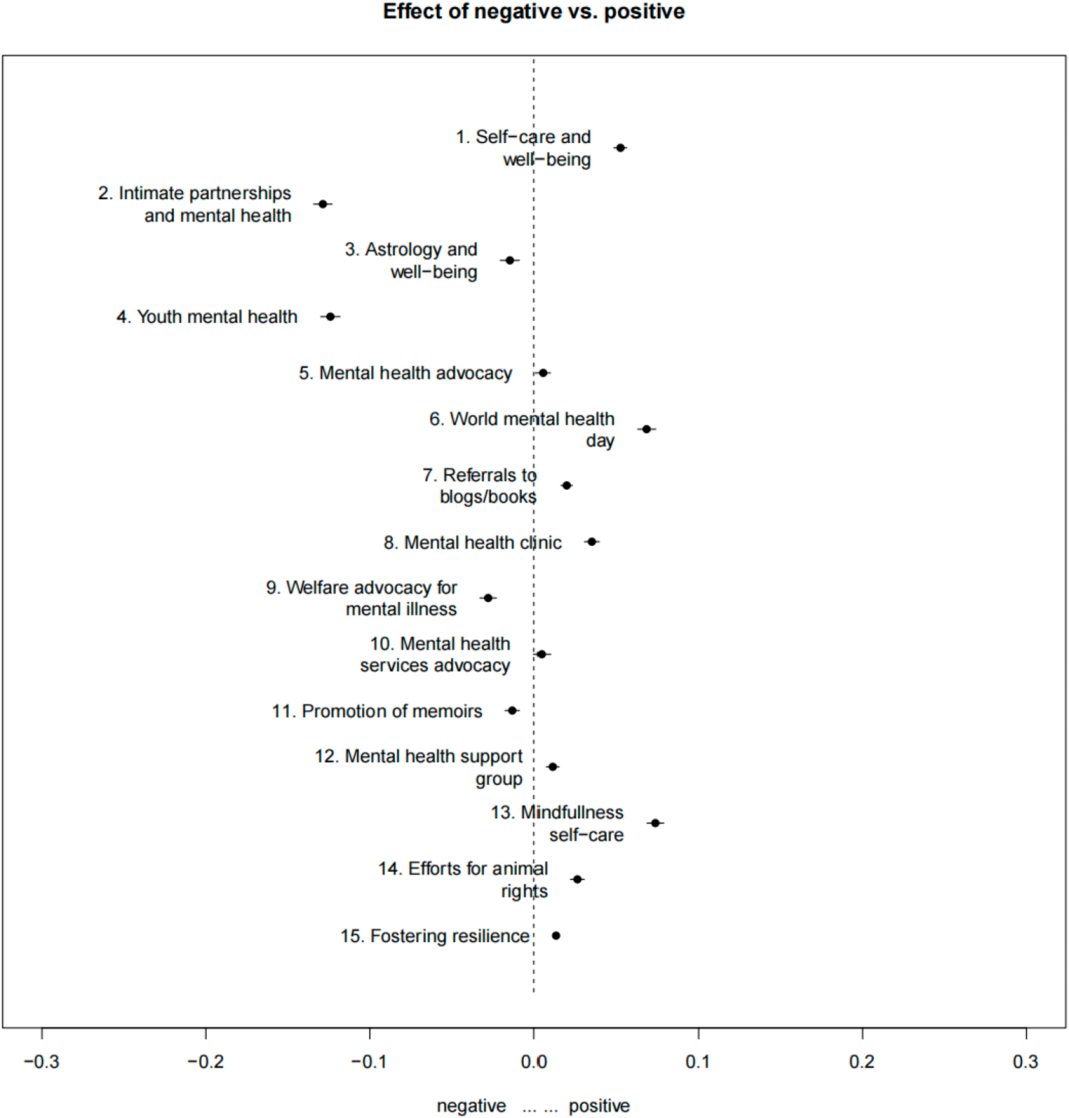
Sentiment-Based Topical Variations in Spanish-Language Twitter Sample, Twitter Academic API Search from 09/19/22 − 10/10/22. NOTE: Every topic is visualized using a horizontal line with a central dot, representing the uncertainty level and coefficient of the sentiment on that topic. When the line does not intersect the central axis (0), it suggests a significant divergence in the topic between positive and negative sentiments. In contrast, if the line intersects the central axis, it implies no substantial difference in the topic between these two sentiments

Figure 1 summarized the results of the regression analysis of all Spanish-language Twitter topics. Positive sentiment was statistically significant for the following topics: Topic 1 self-care and well-being (β = 0.052, *p* < .001), Topic 5 mental health advocacy (β = 0.005, *p* < .05), Topic 6 world mental health day (β = 0.068, *p* < .001), Topic 7 referrals to blogs/books (β = 0.019, *p* < .001), Topic 8 mental health clinics (β = 0.034, *p* < .001), Topic 12 mental health support groups (β = 0.011, *p* < .001), Topic 13 mindfulness self-care (β = 0.074, *p* < .001), and Topic 15 fostering resilience (β = 0.013, *p* < .001). Conversely, negative sentiment was statistically significant for Topic 2 intimate partnerships and mental health (β=-0.128, *p* < .001), Topic 3 astrology and well-being (β=-0.015, *p* < .001), Topic 4 youth mental health (β=-0.124, *p* < .001), Topic 9 welfare advocacy for mental illness (β=-0.027, *p* < .001), and Topic 11 promotion of memoirs (β=-0.013, *p* < .001). No significant sentiment was observed in Topic 10 mental health services advocacy.

### Themes across English-language mental health Twitter hashtags

The English-language tweets comprising the 15 resulting topics were classified into six overarching and distinct themes: awareness, self-care, lived experience, service providers, political activism, and marketing. See Table 3 for high-probability words and example tweets for each of the 15 topics. Within the awareness theme, topics included PTSD and suicide awareness (Topic 6), World Mental Health Day (Topic 7), and comorbidity related to trauma, pain, substance abuse, and cardiovascular disease (Topic 12). The self-care theme encompassed topics such as faith and wellbeing (Topic 3), mental health self-care (Topic 4), mindfulness self-care (Topic 11), workplace mental health (Topic 14), and fostering resilience (Topic 15). Under the lived experience theme, there were topics related to referrals to blogs/books (Topic 8) and the promotion of mental health memoirs (Topic 13). The service providers theme focused on supporting mental health service providers (Topic 2). The political activism theme included topics on mental illness labels for protest (Topic 5) and promotion of white papers (Topic 9). Lastly, the marketing theme comprised topics on mental illness labels for marketing (Topic 1) and complementary and alternative medicine (Topic 10).

**Fig. 2:**
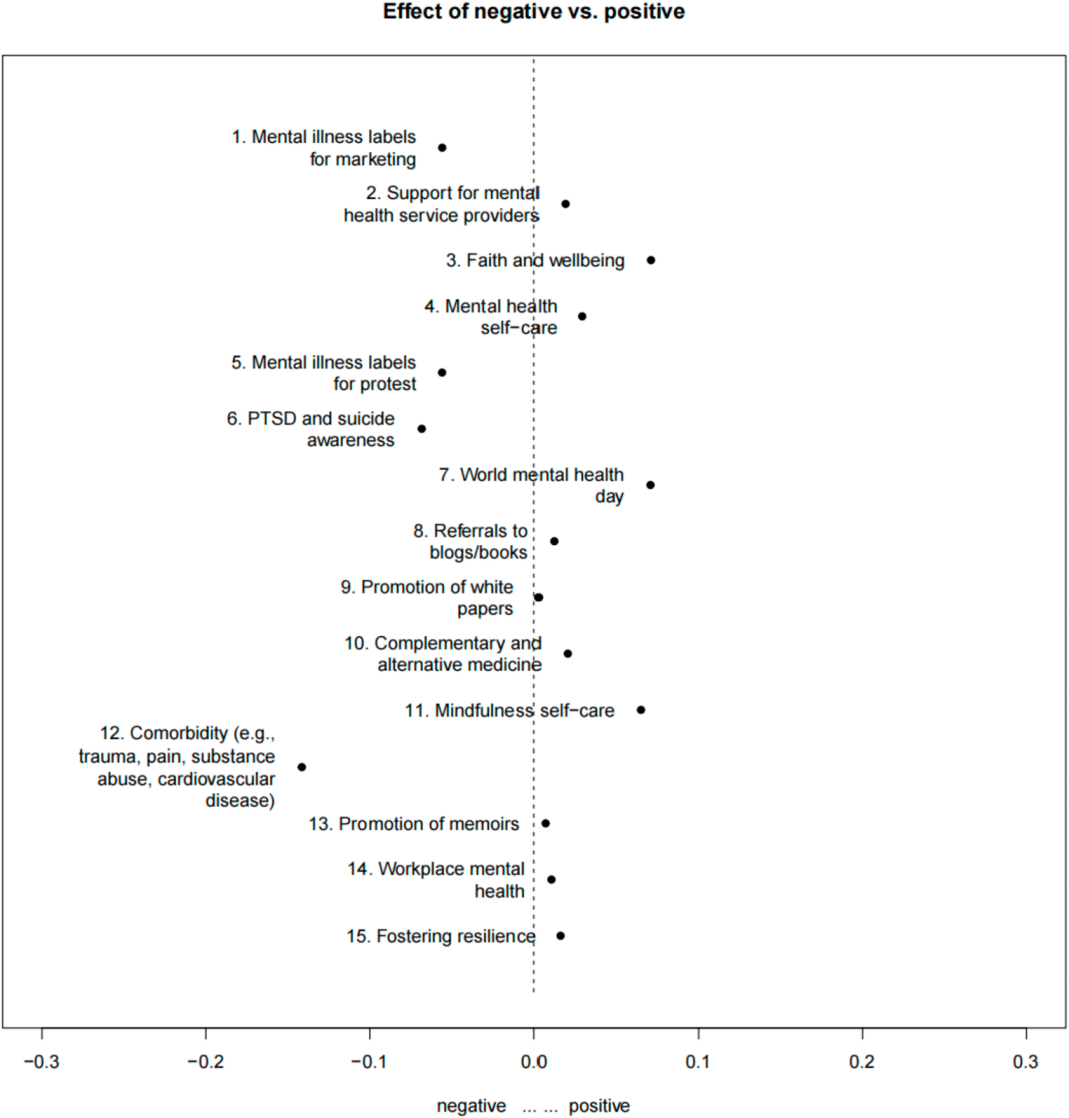
Sentiment-Based Topical Variations in English-Language Twitter Sample, Twitter Academic API Search from 09/19/22 − 10/10/22. NOTE: Every topic is visualized using a horizontal line with a central dot, representing the uncertainty level and coefficient of the sentiment on that topic. When the line does not intersect the central axis (0), it suggests a significant divergence in the topic between positive and negative sentiments. In contrast, if the line intersects the central axis, it implies no substantial difference in the topic between these two sentiments

Regression analysis revealed that the following Topics exhibited a statistically significant presence of positive emotions tweets (see Fig. 2): Topic 2 supporting mental health service providers (β = 0.019, *p* < .001), Topic 3 faith and wellbeing (β = 0.070, *p* < .001), Topic 4 mental health self-care (β = 0.029, *p* < .001), Topic 7 World Mental Health Day (β = 0.070, *p* < .001), Topic 8 referrals to blogs/books (β = 0.012, *p* < .001), Topic 9 promotion of white papers (β = 0.003, *p* < .001), Topic 10 complementary and alternative medicine (β = 0.020, *p* < .001), Topic 11 mindfulness self-care (β = 0.065, *p* < .001), Topic 13 promotion of mental health memoirs (β = 0.007, *p* < .001), Topic 14 workplace mental health (β = 0.010, *p* < .001), and Topic 15 fostering resilience (β = 0.016, *p* < .001). In contrast, the following Topics were significantly associated with negative sentiment tweets (see Fig. 2): Topic 1 mental illness labels for marketing (β=-0.056, *p* < .001), Topic 5 mental illness labels for protest (β=-0.056, *p* < .001), Topic 6 PTSD and suicide awareness (β=-0.068, *p* < .001), and Topic 12 trauma, pain, substance abuse, and cardiovascular disease (β=-0.141, *p* < .001).

## Discussion

Our study results align with our expectations, demonstrating the existence of more mental health awareness content in English-language Twitter than Spanish-language Twitter. Furthermore, this content exhibited higher overall positive sentiment. Both Spanish- and English-language tweets included themes related to awareness, self-care, lived experience, and service providers. However, there were notable distinctions within these themes. Within the awareness theme, Spanish tweets predominantly focused on social- and family-related topics (e.g., intimate partnerships, youth, advocacy, social welfare, and healthcare). In contrast, English tweets focused more on specific mental illness symptoms and labels such as PTSD, suicidality, and comorbidity related to trauma, pain, substance abuse, and cardiovascular disease. Within the self-care theme, Spanish-language tweets emphasized areas such as astrology, mindfulness, and resilience, while English-language tweets focused on religiosity, workplace well-being, mindfulness, and resilience. Tweets in both languages promoted mental health support group blogs and memoirs discussing lived experiences with mental illness, as well as mental health services. However, Spanish-tweets tended to focus on availability of mental health clinics while English-tweets tended to focus on burn out among mental health service providers. Two noteworthy exceptions of English tweets in terms of quality of information concerned the appropriated use of mental health labels and diagnoses to promote a political agenda (i.e., stigmatizing use of a mental illness label to describe disagreement with a politician) and/or market products that may or may not be evidence-based for the treatment of mental illnesses (e.g., complementary/alternative medicine, cannabis-related products).

Mental health awareness campaigns are often spearheaded by social media influencers on Twitter. Some of these influencers may use expertise, training, and experience to lead such influence and power in a public forum that diverges from clinical expertise, training, and experience, possibly leading to different priorities and values concerning population mental health. While mental health awareness messaging can have a positive impact by offering accessible support to individuals and communities grappling with mental illness, offering empowerment and challenge to stigma, these campaigns may also lead to unanticipated consequences including possible adverse effects that warrant consideration. One obvious adverse effect is the unequal distribution of content in terms of quantity and quality across language and cultural social media like Twitter, which tends to disproportionately disadvantage Latino and Spanish-speaking populations. This is largely due to health inequity-producing processes where health and healthcare innovations diffuse to marginalized populations less often and much later [26, 27, 33–35]. Slow dissemination of innovations also applies to mental health (e.g., effective media campaigns about mental health promotion), which may or may not be protective depending on the effectiveness of the mental health media campaign and its evidence-base, if any [36]. On the one hand, mental health awareness campaigns can be mental health promoting by increasing help-seeking intentions, but on the other hand, they may also create adverse effects such as increasing stigmatizing attitudes and behaviors, despite being delivered with pro-social intentions. Still, effectual mental health awareness campaigns (e.g., the new suicide and crisis lifeline #988) that are slow to disseminate lifesaving and life-enhancing information in a timely manner to marginalized populations including Latino populations carries significant risks.

Our study is not without limitations. First, this study relies on a lexicon-based approach for sentiment analysis. This approach does not consider the context in which the text is used such as sarcasm, irony, or pejorative contexts, making interpretation challenging. Meanwhile, the use of pre-defined categories for sentiment analysis may limit our ability to capture nuanced sentiments that are not included in the lexicon or predefined categories. Future research could explore the use of more advanced natural language processing techniques such as deep learning or machine learning algorithms to improve the accuracy and granularity of the results. In addition, follow-up studies could consider incorporating additional data sources such as user socio-demographics or geolocation to gain more context for understanding the tweets and the factors that may influence their sentiment on social media. Exploring other social media platforms such as online forums (e.g., Reddit, Quora) could offer a more comprehensive view of the various language and cultural discourse about mental health including misinformation and disinformation. Despite these limitations, our study demonstrates the lower prevalence of mental health help-seeking resources and recovery framing in Spanish-language social media overall. Future research could investigate other comprehensive datasets to validate our findings.

There is also an urgent need to develop advanced tools to address the gap that we sought to explore. This gap pertains to the lack of research on mental health content on Spanish-language media across emerging technologies, especially in comparison to English-language media using a health equity frame. Future research must explore mental health awareness content across social media in various languages and cultures by using different natural language applications, but this pursuit will be challenging considering that natural language applications tend to be developed in English-language first and then adapted to other languages far too later. In our study, we used an older application available for both Spanish and English languages due to the lag in the development of machine learning applications for Spanish-language content. Moreover, the newest applications are not yet accessible for Spanish-language analysis. Addressing this challenge requires the development of new rigorous approaches to the assessment of social media content that considers global users and equity. Although social media data is privately owned and managed in the tech sector and no national or public archive exists, web-scraping methods for surveillance are available for select data (e.g., Twitter API). However, these data are only available to the extent that the private tech sector allows to be publicly sourced and scraped. It is unknown how these available samples are selected such as if they are random or representative samples for behavioral content analysis. Web-scraping methods are not yet widely available for some popular social media apps, especially for youth (i.e., Instagram, Snapchat), or for capturing content in Spanish or other language and cultural types, limiting both internal and external validity. Furthermore, social media data only offers a broad overview of the topics discussed, without providing in-depth insights into the underlying reasons or motivations for these discussions. Future research could employ mixed methods of quantitative surveys and qualitative methods along with social media content to triangulate methods, explore divergence in results, or examine whether results are robust to the approach.

In sum, mental health messaging on social media is commonly implemented without considering unintended consequences and without any publicly available guidelines about responsible posting of mental health awareness content for social media users. Mental health communication guidelines have been developed for media professionals, especially news journalists, regarding the responsible reporting of high-profile suicides, although these interventions have not fully considered or included Spanish-language media professionals [13]. Nevertheless, similar guidelines do not exist for typical and lay social media users, which is a necessary step for responsible social media posting of mental health content. As we strive to increase the availability of quality mental health information on social media, we should prioritize language and cultural representativeness in the development of such content and the novel tools available to conduct research on this content. Making mental health content more equitable, accessible, and transparent can be a useful strategy to improve population mental health in the United States.

## Data Availability

No datasets were generated or analysed during the current study.

## Footnotes

1 Highest Prob identifies the most probable words, FREX evaluates the significance of a word within the topic relative to its frequency in the corpus, Lift measures the word’s association with the topic, and Score provides an overall importance score for the word within the topic.
